# Unicellular Eukaryotic Community Response to Temperature and Salinity Variation in Mesocosm Experiments

**DOI:** 10.3389/fmicb.2018.02444

**Published:** 2018-10-09

**Authors:** Natassa Stefanidou, Savvas Genitsaris, Juan Lopez-Bautista, Ulrich Sommer, Maria Moustaka-Gouni

**Affiliations:** ^1^Department of Botany, School of Biology, Aristotle University of Thessaloniki, Thessaloniki, Greece; ^2^School of Economics, Business Administration and Legal Studies, International Hellenic University, Thermi, Greece; ^3^Department of Biological Sciences, The University of Alabama, Tuscaloosa, AL, United States; ^4^Geomar Helmholtz Centre for Ocean Research Kiel, Kiel, Germany

**Keywords:** coastal marine plankton, climate change, Illumina sequencing, 18S rRNA gene, cDNA, RNA/DNA ratio

## Abstract

Climate change has profound impacts on marine biodiversity and biodiversity changes in turn might affect the community sensitivity to impacts of abiotic changes. We used mesocosm experiments and Next Generation Sequencing to study the response of the natural Baltic and Mediterranean unicellular eukaryotic plankton communities (control and +6°C heat shock) to subsequent salinity changes (-5 psu, +5 psu). The impact on Operational Taxonomic Unit (OTU) richness, taxonomic and functional composition and rRNA:rDNA ratios were examined. Our results showed that heat shock leads to lower OTU richness (21% fewer OTUs in the Baltic and 14% fewer in the Mediterranean) and a shift in composition toward pico- and nanophytoplankton and heterotrophic related OTUs. Heat shock also leads to increased rRNA:rDNA ratios for pico- and micrograzers. Less than 18% of shared OTUs were found among the different salinities indicating the crucial role of salinity in shaping communities. The response of rRNA:rDNA ratios varied highly after salinity changes. In both experiments the diversity decrease brought about by heat shock influenced the sensitivity to salinity changes. The heat shock either decreased or increased the sensitivity of the remaining community, depending on whether it removed the more salinity-sensitive or the salinity-tolerant taxa.

## Introduction

Multiple components of climate change are projected to affect all levels of biodiversity and thus ecosystems structure and resilience (Bellard et al., 2012). Based on current knowledge (Intergovernmental Panel on Climate Change [IPCC], 2014), sea surface temperature is predicted to increase up to 3–5°C by the year 2100 with even stronger increases in semi-enclosed coastal seas. In addition to increase mean temperature extreme events, such as heat waves, are expected to increase in frequency and strength. Altered regimes of ice-melt, evaporation and precipitation regimes will change salinity (Harley et al., 2006), with a moderate increase of salinity globally by 0.5 psu, but much stronger changes regionally. For the western Baltic Sea, one of our study sites, Andersson et al. (2015) predict a decrease of 2–3 psu during summer season as an ensemble average of several models, while some individual models predict even stronger decreases (Meier et al., 2006). For Thermaikos Bay, Krestenitis et al. (2012) found a salinity decrease of almost 1 psu for the period 1994–2007 with a continuous trend. Physical changes like these will lead to changes in community composition, biodiversity and functional attributes which in turn will influence the responsiveness of communities to further physical changes, as has been conceptualized by the double-stressor concept of Vinebrooke et al. (2004). So far the impact of different aspects of climate change on plankton communities has been studied quite extensively (e.g., Yvon-Durocher et al., 2015; Kaur-Kahlon et al., 2016; Moustaka-Gouni et al., 2016), while the effect of plankton community changes on the sensitivity to environmental pressures has not been the focus of experimental studies.

The use of experimental mesocosms is a well-established tool to explore microbial community responses to environmental changes with the advantage of manipulation, high replication and free factorial combination of stress factors (Benton et al., 2007). Until recently, the majority of investigations that studied the impacts of climate change on microeukaryotic planktic community composition were based on morphological species determination by microscopy. This provides a sufficient resolution in taxa rich in distinctive morphological features (e.g., diatoms, thecate dinoflagellates) while it might underestimate actual biodiversity in taxa poor in distinctive morphological features (e.g., naked flagellates) and miss rare, small, and cryptic or endosymbiotic species (Medinger et al., 2010; Behnke et al., 2011; Christaki et al., 2014). Investigations of the 18S rRNA gene (rDNA herein) diversity often reveal long lists of previously undiscovered taxa of unicellular eukaryotes (Bik et al., 2012). Next Generation Sequencing (NGS) provides a comprehensive tool for investigations of marine microeukaryotic community structure and has recently revealed previously hidden biodiversity (de Vargas et al., 2015). The majority of mesocosm experiments using NGS studied the impacts of climate change on bacterial communities via 16S rDNA sequencing (Muscarella et al., 2014; Baltar et al., 2016; Guo et al., 2016) and only few examined planktic microeukaryotic assemblages (Belkin et al., 2015; Moustaka-Gouni et al., 2016).

While sequencing of rDNA allows detecting the majority of microbe biodiversity regardless of their viability and inferred activity, estimating microbial activities is fundamental to understand the functioning of ecosystems. Generally the quantity of rRNA is proportional to both the number of ribosomes and total RNA concentration (Poulsen et al., 1993), therefore it can be used as an indicator of cell activity. In recent years, the advance of NGS tools has allowed the combination of rDNA sequencing along with rRNA sequencing (RNA-seq, by means of cDNA sequencing) as a way to distinguish organisms with strong indication of high ribosomal activity (henceforth active) from those with low/no ribosomal activity (henceforth non-active) (Campbell et al., 2011; Massana et al., 2015; Hu S.K. et al., 2016). Thus, the ratio of rRNA sequences to rDNA sequences (rRNA:rDNA) has been used as a proxy of metabolic activity (Logares et al., 2015; Hu S.K. et al., 2016). This application has revealed a significant portion of rare species with high activity (Campbell et al., 2011; Hu S.K. et al., 2016) that could act as a genetic reservoir and respond rapidly to environmental changes (Logares et al., 2015).

In mesocosm experiments, phylogenetic markers have been used with classical molecular tools in order to identify environmental variability effects on bacterial (e.g., Lebaron et al., 1999; van der Zaan et al., 2010; Newbold et al., 2012) archaeal (e.g., Bowen et al., 2013), and protistan (e.g., Countway et al., 2005) communities. Here we chose to apply, for the first time in a mesocosm experiment, 18S rDNA sequencing together with 18S rRNA high throughput sequencing to study the impacts of heat shock and changing salinity on composition and inferred activity of two natural microeukaryotic planktic communities. The two study sites were Kiel Bight in Baltic Sea and Thermaikos Bay in Mediterranean Sea. Kiel Bight is a shallow ending of the Baltic Sea experiencing a wide range of salinity throughout the year. It is a eutrophic marine ecosystem where phytoplankton biomass has been doubled the last century (Wasmund et al., 2008). In the North-Eastern Mediterranean, Thermaikos Bay is the innermost part of Thermaikos Gulf, which is a eutrophic coastal area (Simboura et al., 2016) with marked diatom and dinoflagellate blooms (Nikolaides and Moustaka-Gouni, 1990; Mihalatou and Moustaka-Gouni, 2002).

Following Vinebrooke’s et al. (2004) double stressor concept we performed two mesocosm experiments, in which a transient “pulse” stressor should change plankton composition and biodiversity and thus affect the resistance to a subsequent “press” stressor of different nature. For the “pulse” stressor we chose a temperature increase mimicking natural heat-waves, while a positive and negative change in salinity were chosen as subsequent, second stressor.

Our experiments were primarily designed as “proof-of-principle” experiments testing Vinebooke’s double stressor concept. However, we also aimed to put the nature and magnitude of the stressors applied into the context of Global Change research, because the double stressor concept is particularly important in that context, if biodiversity losses driven by one Global Change related factor (e.g., temperature) weaken the resistance of ecological communities against further stressors. The magnitude of temperature change (6°C) employed in our study is clearly below the annual temperature amplitude of mid-latitudes, but conforms to the more pessimistic Global Change scenarios for mean temperature changes in coastal seas and to short term temperature changes during heat-waves which are expected to increase in frequency and magnitude with ongoing climate warming (Intergovernmental Panel on Climate Change [IPCC], 2014). In terms of their temporal scale, the experiments mimick, how a change in the species inventory driven by a heat wave would influence the ability of the resultant phytoplankton communities to cope with salinity changes. The magnitude of salinity changes was within the range of short-term moving of oceanographic fronts of coastal seas and within the predicted range for long-term changes in coastal seas (Meier et al., 2006; Andersson et al., 2015).

Our working hypotheses were:

(1) The heat shock and salinity changes will lead to composition shifts and biodiversity changes of unicellular eukaryotic communities
(2) The heat shock and salinity changes will affect rRNA/rDNA ratios of unicellular eukaryotic communities
(3) The heat shocked communities will have lower resistance to salinity changes

## Materials and Methods

### Experimental Design

We performed a two-step experiment. In the first step, experimental plankton communities (10 L each) were subjected to two different temperatures (A: control, i.e., ambient temperature of the study site, H: heated by 6°C) to manipulate biodiversity by temperature stress and use the resultant communities as inocula for the second step, the analysis of responses to salinity. First, autumn plankton from Kiel Bight, Baltic Sea (sampled on 28 October 2015) and early summer plankton from Thermaikos Bay, Mediterranean Sea (sampled on 16 June 2015) were taken and metazoan grazers were removed by sieving through a 200 μm mesh size gauze. Temperature in the heated treatments was increased gradually over 3 days (+2°C per day) and was kept at +6° above ambient for another 3 days. The resultant A_i_ and H_i_ communities where used as inoculum for the second step. Using a single unit for each temperature pre-treatment assured identical starting conditions between the different mesocosms for the subsequent salinity experiment. For the Baltic Sea experiment 7.5 ml of the A_i_ were added to each mesocosm of the A community and 10 ml of the H_i_ to each mesocosm of the H community; for the Mediterranean experiment 9.1 ml of the A_i_ were added to each mesocosm of the A community and 10 ml of the H_i_ to each mesocosm of the H community. The second step consisted of 24 outdoor mesocosms (two experimental communities preconditioned by different temperatures × three salinity levels × four replicates, **Table 1**) with a volume of 15 L each. Mesocosms were filled with sterile filtered seawater (0.2 μm) from the study and were inoculated with the A_i_ and H_i_ communities. Inoculum volumes were adjusted to provide equal initial biomasses in all treatments. The experiments were terminated after all treatments reached stationary phase. For sample identification we use a code indicating the temperature pre-treatment (A_i_ and H_i_, i subscript corresponds to start samples of the salinity treatment) and the salinity treatment (f-, f, and f+ for low, ambient and high salinity; f subscript corresponds to end-point samples of the salinity treatment). Thus, A_i_ and H_i_ are at the same time the end-point samples for the temperature pre-treatment and the start samples for the salinity treatment, A_f_, A_f+_, A_f-_, H_f_, H_f+_, and H_f-_ are the end point samples for the salinity treatments.

**Table 1:** General description of A and H communities treatments, and number of DNA and RNA-based OTUs for each experiment.

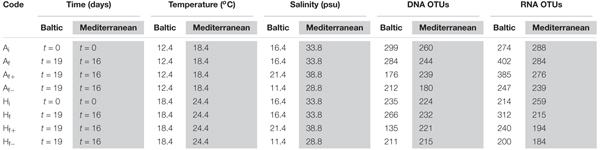

*Values for the Mediterranean experiment are shaded.*

### Sample Collection

Water temperature, salinity and fluorescence (fluorometer 10-AU, Turner Design) in the Baltic mesocosms were measured every second day during the first week and every day after the first week of the experiment. In the Mediterranean experiment, phytoplankton abundance was measured instead of fluorescence using the inverted microscope method (Utermöhl, 1958). At the end of the experiments, subsamples (48 samples) were analyzed with the inverted microscope and morphospecies richness, abundance and biomass showed no significant differences among the replicates according to Kolmogorof–Smirnov statistical test. For the molecular analysis, subsamples of 100 mL were taken from each replicate for DNA and RNA sequencing before the inoculation and at the end of the salinity experiment. After collection, DNA and RNA samples were filtered immediately through 0.2 μm nucleopore filters (47 mm diameter) using very low filtration pressure. The filters were stored at -80°C until nucleic acid extraction.

### DNA and RNA Extraction

Filters of the same replicates were pooled together before DNA and RNA extraction, based on microscopy data that showed no significant differences between replicates in terms of morphospecies richness, abundance and biomass. Nucleic acid extractions were done using PowerWater DNA isolation kit (Mo Bio Laboratories Inc., Carlsbad, CA, United States) and PowerSoil RNA isolation kit (Mo Bio Laboratories Inc., Carlsbad, CA, United States), following the manufacturers protocol. Minor modifications included introduction of three cycles of freeze-thaw (-80°C, 5 min, 65°C, 5 min) and beat beading with 2 × 5 min intervals on a horizontal vortex. The RNA samples were tested for the presence of DNA contamination by PCR and then reversed-transcribed using qPCRBIO cDNA Synthesis Kit (PCR Biosystems, United Kingdom). The nucleic acid samples contained between 3.2 ng u L^-1^ and 25.8 ng u L^-1^ of total DNA and between 3.1 ng u L^-1^ and 16.4 ng u L^-1^ of total RNA as measured by NanoDrop (Thermo Scientific, United States).

### PCR and Tag Pyrosequencing

Tag pyrosequencing of 18S rDNA was performed using PCR amplification of the V2 and V3 region and by using the two eukaryotic primers 18S-82F (5′-GAAACTGCGAATGGCTC-3′) (Lopez-Garcia et al., 2003) and Euk-516r (5′-ACCAGACTTGCCCTCC-3′) (Amann et al., 1990). These primers were designed to amplify 470–480 bp in the region. Furthermore, cDNA (representing RNA) was amplified using the same primer pair targeting the same region. PCR reactions and barcode amplicon sequencing process described by Dowd et al. (2008) was performed by the Mr. DNA Company (Shallowater, TX, United States)^1^. Briefly, PCR conditions included a denaturation step at 95°C for 5 min, 30 cycles of amplification that were performed at 95°C for 30 s, 50°C for 30 s and 72°C for 1 min. A final extension step was performed for 7 min at 72°C. Subsequently, PCR products were purified using calibrated Ampure XP beads and the purified products were used to prepare the DNA libraries by following the Illumina MiSeq DNA high-throughput library preparation protocol. DNA library preparation and sequencing was performed at Mr. DNA (Shallowater, TX, United States)^2^ on a MiSeq following the manufacturer’s guidelines. Sequences were submitted to GenBank-SRA under the accession number SRX3222420.

### Read Processing

The produced reads were processed using MOTHUR v 1.34.0 software following the standard operating procedure (Schloss et al., 2009, 2011). Briefly, forward and reverse reads were joined, and the barcodes were removed. Reads < 200 bp, with homopolymers >8 bp or with ambiguous base calls were removed from downstream analysis. The remaining reads were dereplicated to the unique sequences and aligned independently against SILVA 128 database, containing 140,020 eukaryotic SSU rRNA sequences (Quast et al., 2013). Then, reads suspected of being chimeras were removed using the UCHIME software (Edgar, 2010). The data sets were normalized to the sample with the lowest number of reads using the subsample command in MOTHUR, so that rDNA samples contained 41,504 reads and rRNA samples 61,910 reads. These reads were clustered into OTUs at 97% sequence similarity threshold. Singletons were removed, as they were likely erroneous sequencing products (Kunin et al., 2010; Behnke et al., 2011). After the analyses, 2948 OTUs in rDNA samples and 2947 OTUs in rRNA samples were produced in total, and were taxonomically classified using BLASTN (Altschul et al., 1990) on the PR2 curated database, which contains protists sequences (Guillou et al., 2013). OTUs that were affiliated to Metazoa (eggs, larvae, fragments of animals) were removed, thus 1715 OTUs belonging to protists remained in rDNA samples and 1653 OTUs in rRNA samples.

### Assignment of Unicellular Eukaryotes in Functional/Trophic Groups

All OTUs were assigned to six trophic groups according to their trophic roles (**Supplementary Table 1**), autotrophs (e.g., Bacillariophyceae), mixotrophs (e.g., Haptophyceae), micrograzers (e.g., Amoebozoa), picograzers [e.g., Marine stramenopile (MAST)], parasites [e.g., Marine alveolates (MALVs)] and decomposers (e.g., Fungi). Briefly, each OTU was individually examined and assigned to one of the six groups after gathering the current available information for it. Microscopic observations (phase contrast and epifluoresence microscopy) were also used for the classification and assignment of every microscopically detectable OTU (e.g., *Skeletonema*, *Prorocentrum*, *Imantonia*). For the rest OTUs which were undetectable with microscopic techniques (e.g., MALVs, *Pirsonia*), the assignment was done according to extensive literature search (**Supplementary Table 1**). For instance, MALVs and *Pirsonia* are classified as parasites (Skovgaard, 2014) and Picobilyphyta are classified as picograzers (Moreira and López-García, 2014).

### Data Analysis

Rarefaction curves for all samples were calculated with the PAST 2.17c software, in order to determine if the sequencing effort was sufficient to fully assess the OTUs richness. The ratios of auto- and mixotrophic to heterotrophic OTUs (S_auto_/S_het_) and auto- and mixotrophic to consumers number of OTUs (S_auto_/S_grazers + parasites_) were calculated as metrics related to the community trophic structure. Decomposers were excluded from the calculation of the S_auto_/S_grazers + parasites_ ratio as they did not consume living biomass. OTUs were grouped into two categories according to their relative abundance. Per sample abundant OTUs were defined as those with relative abundance > 1% of the total number of reads in the sample while per sample rare as those with relative abundance < 0.2% (Mangot et al., 2013; Logares et al., 2014; Genitsaris et al., 2015). For defining overall abundant and rare OTUs the aforementioned thresholds were divided by a factor of 10; overall abundant OTUs were represented with >0.1% of the total number of reads while overall rare with <0.02% of the total number of reads (Logares et al., 2014; Genitsaris et al., 2015). The relationship between 18S rRNA and 18S rDNA frequency was examined for each common OTU in the two datasets of rDNA and rRNA. rRNA:rDNA ratios were used as a proxy in order to examine the relative activity of the obtained OTUs. First, the range of rRNA:rDNA ratio was calculated for the taxonomic groups and then the average rRNA:rDNA ratio for each taxonomic and for each trophic group was calculated across the treatments in order to evaluate the alterations in relative activity in relation to temperature and salinity changes. The resistance of A and H communities to salinity change was measured by log-response ratio [LR = ln(Y1/Y0)], where Y1 is the metric of choice in the treatments (A_f-_, A_f+_, H_f-_, H_f+_) and Y0 the metric of choice in the control (A_f_, H_f_). A LR close to zero indicates little change, while strongly positive or negative ratios indicate strong change. As metrics of response, we chose the two most important measures of diversity, OTU-richness (S), and evenness (E) (Wilsey and Potvin, 2000) and two measures of trophic structure, S_auto_/S_het_ and S_auto_/S_grazers+parasites_.

### Statistical Analysis

Non-parametric Kolmogorov–Smirnov paired test (K–S test) was used to evaluate whether the rDNA and rRNA relative abundance between A_i_ and H_i_ unicellular eukaryotic communities are equal. The equality among the rDNA and rRNA relative abundance across the three salinities was examined with non-parametric Kruskal–Wallis test. The significance of heat shock and salinity changes effect on taxonomic composition across the treatments was tested with Pearson’s chi-square test. Kendall rank correlation coefficient was used to measure the ordinal association between 18S rDNA and 18S rRNA relative abundance. Finally, we used multidimensional scaling (MDS) based on Jaccard similarity coefficient, due to its sensitivity for the rare taxa (Chao et al., 2005), to compare the community composition.

## Results

### rDNA and rRNA Community Structure

**FIGURE 1:**
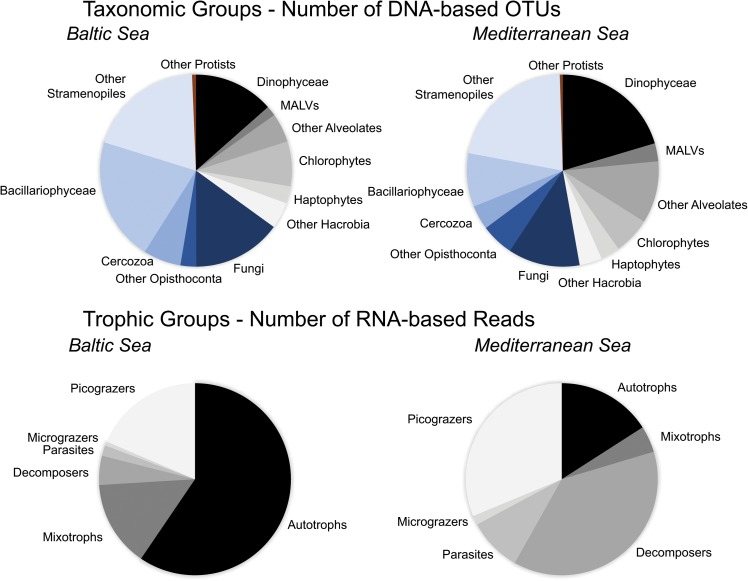
Pie charts of the number of DNA-based OTUs of taxonomic groups, and number of RNA-based reads of trophic groups detected in the Baltic Sea and Mediterranean Sea experiments.

A total of 1715 unique OTUs, were identified over all samples after read denoising, chimera removal, removal of metazoan OTUs and normalization. These were affiliated to 9 protistan supergroups (Alveolates, Amoebozoa, Apusozoa, Excavata, Archeoplastida, Hacrobia, Opisthoconta, Rhizaria, Stramenopiles) and were further divided into 36 taxonomic groups. Two hundreds and thirty-seven OTUs were shared in both experiments while 639 OTUs were observed only in the Baltic experiment and 839 OTUs only in the Mediterranean experiment. Among the 36 taxonomic groups in the Baltic experiment, Bacillariophyceae had the highest number of OTUs (382) followed by Fungi (275 OTUs) and Dinophyceae (248 OTUs) (**Figure 1**). Regarding the trophic groups, autotrophs had the highest OTU richness (374) followed by picograzers (166 OTUs) and parasites (124 OTUs) (**Supplementary Table 2B**). In the Mediterranean experiment, Dinophyceae was the taxonomic group with the highest number of OTUs (403) followed by Fungi (239 OTUs) and Bacillariophyceae (178 OTUs) (**Figure 1**) and mixotrophs were the trophic group with the highest OTU richness (298) followed by picograzers (270 OTUs) and parasites (212 OTUs) (**Supplementary Table 2B**). The RNA dataset was composed by 1653 unique OTUs. In the Baltic experiment, Bacillariophyceae prevailed as the most abundant taxonomic group (289,044 reads-56%) followed by Haptophyceae (74,696 reads-15%) and Fungi (25,128 reads-5%) (**Supplementary Table 2A**). Autotrophs were the most abundant trophic group (304,766 reads-59%) followed by picograzers (95,264 reads-19%) and mixotrophs (74,443 reads-14%) (**Figure 1**). In the Mediterranean experiment, Fungi were the most abundant taxonomic group (182,643 reads-38%) followed by Dinophyceae (48,784 reads-10%) and Haptophyceae (21,723 reads-5%) (**Supplementary Table 2A**) and decomposers were the most abundant trophic group (180,643 reads-38%) followed by picograzers (149,458 reads-31%) and autotrophs (76,153 reads-16%) (**Figure 1**). Finally, rarefaction curves reached a plateau in all cases when ≥97% cutoff levels of read similarities were applied (**Supplementary Figure 1**) both in DNA and RNA analyses.

### Effects of Changing Salinity on A and H Microeukaryotic Community Structure

**FIGURE 2:**
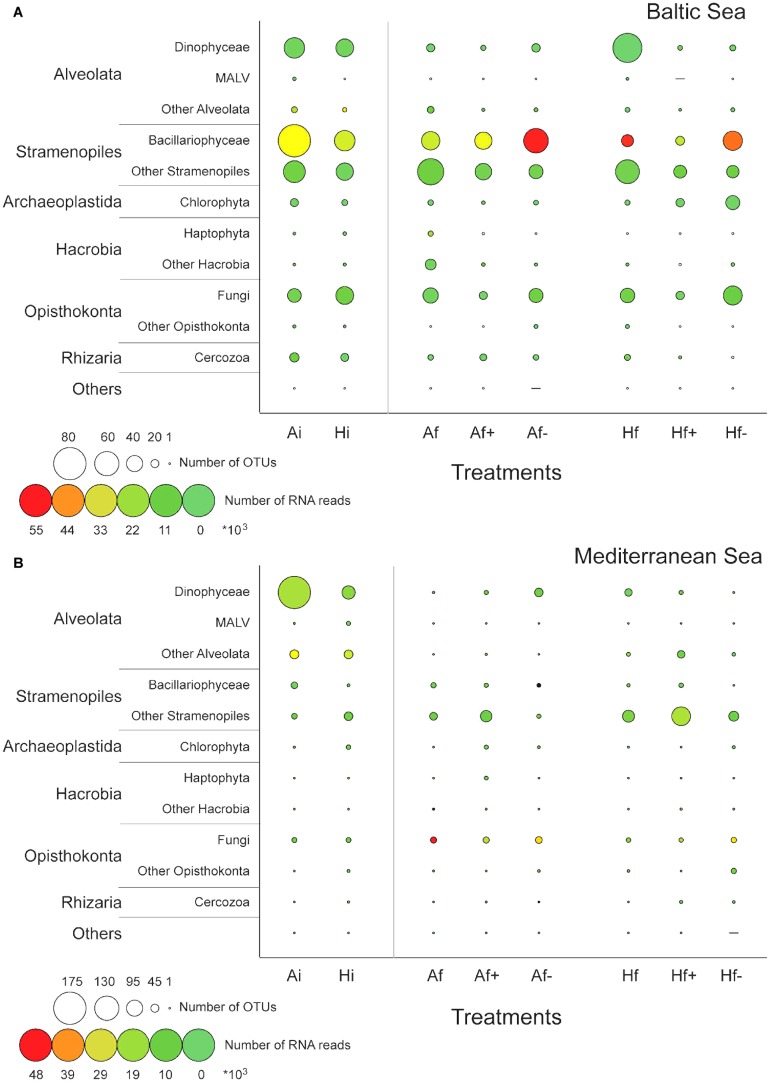
Variation of the number of DNA-based OTUs (circle diameter) and the relative abundance (circle color) of RNA-based reads belonging to the dominant high-level taxonomic groups in the Baltic experiment **(A)** and in the Mediterranean experiment **(B)**. The taxonomic affiliation was based on BLASTN searches against the PR2 database. A_i_, initial sample and ambient temperature; A_f_, ambient temperature and salinity; A_f+_, ambient temperature and high salinity (+5 psu); A_f-_, ambient temperature and low salinity (-5 psu); H_i_, initial sample and heat shock (+6^°^C); H_f_, heat shock (+6^°^C) and ambient salinity; H_f+_, heat shock (+6^°^C) and high salinity (+5 psu); H_f-_, heat shock (+6^°^C) and low salinity (-5 psu).

**FIGURE 3:**
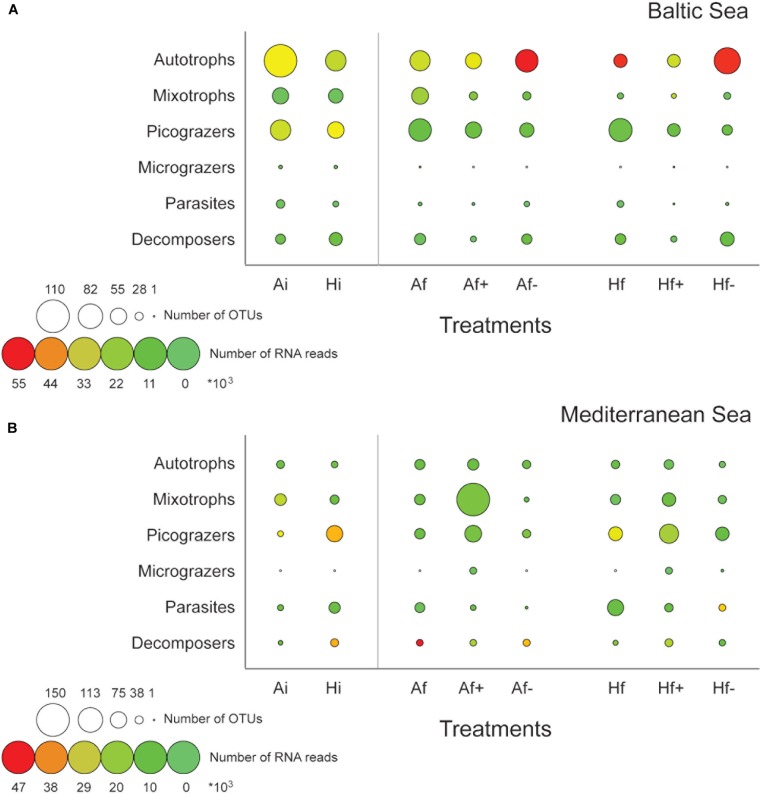
Variation of the number of DNA-based OTUs (circle diameter) and the relative abundance (circle color) of RNA-based reads belonging to the six trophic groups in the Baltic experiment **(A)** and in the Mediterranean experiment **(B)**. The assignment to the trophic groups was based on microscopic observations and literature data. A_i_, inoculum and ambient temperature; A_f_, ambient temperature and salinity; A_f+_, ambient temperature and high salinity (+5 psu); A_f-_, ambient temperature and low salinity (-5 psu); H_i_, inoculum and heat shock (+6^°^C); H_f_, heat shock (+6^°^C) and ambient salinity; H_f+_, heat shock (+6^°^C) and high salinity (+5 psu); H_f-_, heat shock (+6^°^C) and low salinity (-5 psu).

According to K–S paired test equality between the A_i_ and H_i_ communities was rejected in both experiments (*P*_same_> 0.05) and according to chi-square test the effect of the heat shock on community composition was statistically significant in both cases (*P*_Pearson′s_ < 0.05). Totally, in the Baltic experiment 299 DNA-based OTUs were identified in A_i_ community and 235 in H_i_ community and in the Mediterranean experiment 260 in A_i_ and 224 in H_i_ (**Table 1**). Eighty-one Bacillariohyceae related DNA-based OTUs were recorded in A_i_ in the Baltic experiment while only 50 in H_i_ (**Figure 2A**). Their RNA-based relative abundance decreased from 48% in A_i_ to 41% in H_i_ (**Figure 2A**). Autotrophic DNA-based OTUs and RNA-based reads decreased from 109 and 50% in A_i_ to 69 and 42% in H_i_ (**Figure 3A**). In the Mediterranean experiment A_i_ contained 175 Dinophyceae related DNA-based OTUs while H_i_ only 72 (**Figure 2B**). Their RNA-based relative abundance deceased from 30% in A_i_ to 23% in H_i_ (**Figure 2B**). Autotrophs together with mixotrophs had 20 less DNA-based OTUs and 18% less RNA-based reads in H_i_ than in A_i_ (**Figure 3B**). On the other hand, a relatively low increase of Chlorophyceae and Haptophyceae related DNA-based OTUs was detected in H_i_ in both experiments (**Figures 2A,B**). Furthermore, increased DNA-based OTU richness and RNA-based relative abundance was found for Fungi and decomposers in H_i_ in the Baltic experiment and for MAST and picograzers in the Mediterranean experiment (**Figures 2A,B**, **3A,B**). The rest of the taxonomic and trophic groups did not show considerable differences. As less autotrophs and more heterotrophs and parasites were found in H_i_ in both experiments, the ratios S_auto_/S_het_ and S_auto_/S_grazers+parasites_ were lower than in A_i_ (**Supplementary Table 3**).

Bacillariophyceae-related OTUs in the Baltic experiment had the highest DNA-based diversity and RNA-based relative abundance in A_f-_ and in H_f-;_ 20 more DNA-based OTUs were found in A_f-_ and in H_f-_ compared to A_f_ and H_f_ respectively and 40% higher RNA-based relative abundance in A_f-_ than A_f_ and 2% higher in H_f-_ than H_f_ (**Figure 2A**). The highest autotrophic DNA-based OTU richness and RNA relative abundance were observed also in A_f-_ and H_f-_; 10 more DNA-based OTUs and 39% more RNA-based reads were recorded in A_f-_ compared to A_f_ and 20 and 1.5% more in H_f-_ compared to H_f_ (**Figure 3A**). High salinity had little effect on Bacillariophyceae and autotrophs as the DNA-based OTUs and the RNA-based reads between A_f_ and A_f+_ and H_f_ and H_f+_ were similar (**Figures 2A**, **3A**). The highest values of the S_auto_/S_het_ and S_auto_/S_grazers+parasites_ ratios were also calculated in A_f-_ and in H_f-_ while they were similar between A_f_ and A_f+_ and almost similar between H_f_ and H_f+_ (**Supplementary Table 3**). In the Mediterranean experiment salinity changes affected Dinophyceae differently depending on heat shock application. Dinophyceae had 20 more DNA-based OTUs and 8% more RNA-based reads in A_f+_ and A_f-_ than A_f_ but they had 29 less DNA-based OTUs but still 3% more RNA-based reads in H_f+_ and H_f-_ than H_f_ (**Figures 2B**, **3B**). The highest autotrophic and mixotrophic DNA-based OTU richness and RNA-based relative abundance was observed in A_f+_ and the lowest in H_f-_ (**Figure 3B**). S_auto_/S_het_ and S_auto_/S_grazers+parasites_ ratios increased in A_f+_ and H_f+_ compared to A_f_ and H_f_ and remained almost stable between A_f-_ and A_f_ and H_f-_ and H_f_ (**Supplementary Table 3**).

A low number of shared DNA-based OTUs was found among the different salinity treatments of each community in both experiments. In the Baltic experiment 80 shared OTUs out of 452 were found in the A community and 63 out of 425 were found in the H community while in the Mediterranean experiment 60 out of 387 and 89 out of 540 were found respectively (**Supplementary Figure 2**). The Kruskal–Wallis test indicated that there was no equality among the different salinity treatments (*P* > 0.05) in both experiments. However, according to the chi-squared test, the effect of salinity changes to community composition was statistically not significant (*P*_Pearosn′s_ > 0.05). Fungal OTUs together with Bacillariophyceae and Dinophyceae made up the 46% of the shared OTUs in A community in the Baltic experiment and 58% in Mediterranean experiment while the rest of the taxonomic groups contributed with <10% each. In terms of DNA-based relative abundance the genera *Kappamyces* (98% similarity), *Skeletonema* (100% similarity) and *Gyrodinium* (100% similarity) in the Baltic experiment and genera of the class Exobasidiomycetes (100% similarity), the genera *Gyrodinium* (100% similarity) and *Chaetoceros* (100% similarity) in the Mediterranean experiment were dominant shared OTUs. In the H community, OTUs related to Bacillariophyceae, to Dinophyceae and to Chlorophyceae made up 42% of the shared in the Baltic experiment while in the Mediterranean experiment Fungi, Dinophyceae and Bacillariophyceae made up 39% of the shared. OTUs associated with the genera *Skeletonema* (100% similarity), *Gymnodinium* (100% similarity) and *Desmochloris* (100% similarity) in the Baltic experiment and with the genera of the class Exobasidiomycetes (100% similarity), and the genera *Scrippsiella* (98% similarity) and *Chaetoceros* (100% similarity) in the Mediterranean experiment dominated in DNA-based relative abundance among the shared.

**FIGURE 4:**
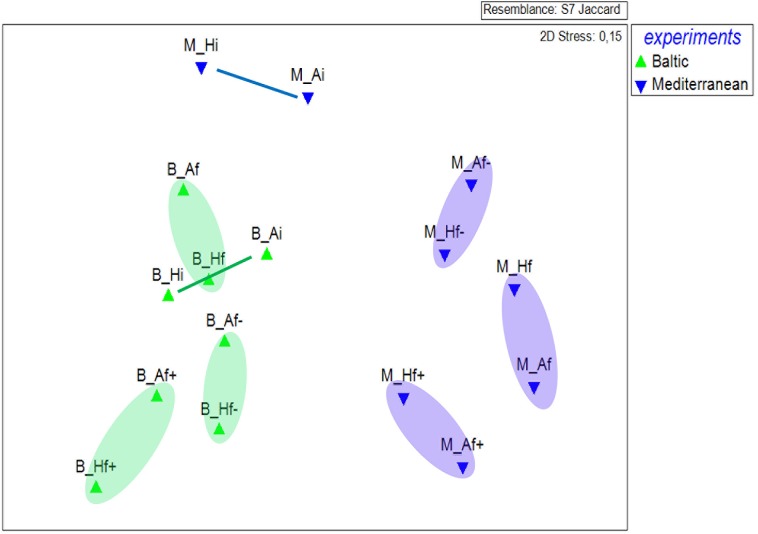
Multidimensional scaling plot of read abundance variation in the different treatment assemblages, according to Jaccard similarity index, of the Baltic (B) and Mediterranean (M) unicellular plankton communities. A_i_, inoculum and ambient temperature; A_f_, ambient temperature and salinity; A_f+_, ambient temperature and high salinity (+5 psu); A_f-_, ambient temperature and low salinity (-5 psu); H_i_, inoculum and heat shock (+6^°^C); H_f_, heat shock (+6^°^C) and ambient salinity; H_f+_, heat shock (+6C) and high salinity (+5 psu); H_f-_, heat shock (+6^°^C) and low salinity (-5 psu).

MDS showed a clear separation of plankton community composition between the two experiments (**Figure 4**). In the Mediterranean experiment, A_i_ and H_i_ communities exhibited major changes from the start to the end of the salinity treatments experiment whereas smaller changes were detected in the Baltic experiment. Nevertheless, salinity treatments in both experiments drove OTU composition in the same direction, irrespective of the temperature pre-treatment. This means, that for example pre-heated and unheated high salinity experimental communities became more similar among each other than experimental units with the same temperature pre-treatment but different salinity treatment.

### Relative Abundance and Inferred Activity of Taxonomic and Trophic Groups

The proportions of rare and abundant OTUs were quite similar across the samples and rare OTUs dominated in both data sets (DNA-based and RNA-based) and in both experiments (**Supplementary Table 4**). Rare DNA-based OTUs in the Baltic experiment fluctuated between 83.9 and 88.4% of the total number of OTUs and rare RNA-based OTUs between 88 and 94.5% while abundant DNA-based OTUs fluctuated between 3.7 and 10.2% and abundant RNA-based OTUs between 1.5 and 4.6%. Similarly, in the Mediterranean experiment rare DNA-based OTUs ranged from 77.4 to 91.7% and rare RNA-based OTUs from 79.4 to 88.8%. Abundant DNA-based OTUs ranged from 3.3 to 10.9% and abundant RNA-based OTUs from 3.5 to 8.3%. The abundant OTUs were affiliated mainly to 8 taxonomic groups (Dinophyceae, Bacillariophyceae, Haptophyceae, Chlorophyceae, Ciliophora, Fungi, Cercozoa and MAST).

In both experiments, the rRNA:rDNA relative abundance relationship revealed a positive correlation [Kendall correlation coefficient (τ_Baltic_) = 0.286, τ_Mediterranean_ = 0.328; *P* < 0.001] suggesting that the abundant OTUs in the DNA dataset were among the most abundant in RNA dataset and vice versa (**Supplementary Figure 3**). However, there were OTUs whose RNA relative abundance did not follow their DNA relative abundance. OTUs below the 1:1 line had a high DNA relative abundance but low RNA relative abundance (e.g., *Heterocapsa*, *Gyrodinium*, *Desmochloris* in the Baltic and *Pelagodinium*, *Pelagostrobilidium*, *Chaetoceros* in the Mediterranean) and OTUs above 1:1 line had low DNA relative abundance but high RNA relative abundance (e.g., *Imantonia*, *Telonema* in the Baltic and *Phaeosphaeria*, genera of the class Exobasidiomycetes in the Mediterranean).

The overall range of average RNA:DNA ratio showed large variability among taxonomic groups. The highest variation in the Baltic experiment was observed for Amoebozoa, Hacrobia excluding Haptophyceae (Centrohelioza, Cryptophyceae, Katablepharidota, Picobiliphyta, Telonemia), Apusozoa and Ciliophora and in the Mediterranean experiment for Amoebozoa, Fungi and Haptophyceae (**Supplementary Figure 4**). On the contrary, the range of the estimated RNA:DNA ratio for Dinophyceae, Bacillariophyceae and Chlorophyceae and Bacillariophyceae, MALVs and Apusozoa respectively remained low.

**FIGURE 5:**
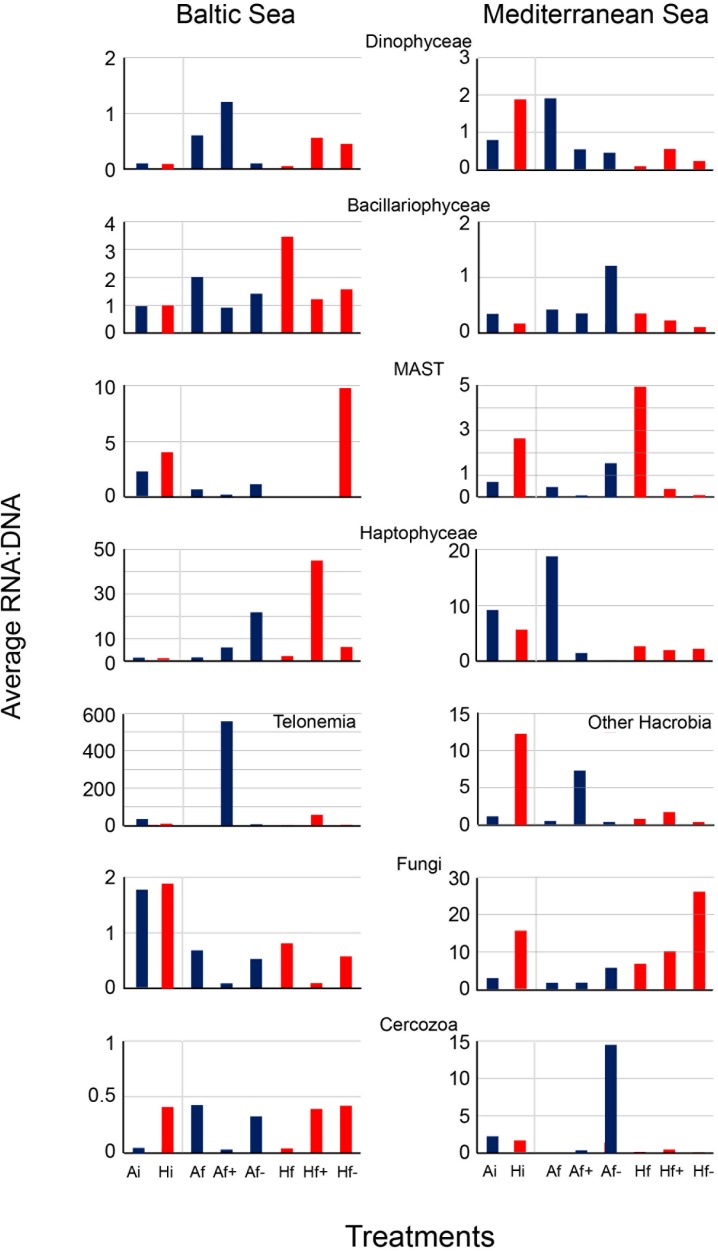
Average RNA:DNA ratios from dominant taxonomic groups in the Baltic experiment and in the Mediterranean experiment. A_i_, inoculum and ambient temperature; A_f_, ambient temperature and salinity; A_f+_, ambient temperature and high salinity (+5 psu); A_f-_, ambient temperature and low salinity (-5 psu); H_i_, inoculum and heat shock (+6^°^C); H_f_, heat shock (+6^o^C) and ambient salinity; H_f+_, heat shock (+6^°^C) and high salinity (+5 psu); H_f-_, heat shock (+6^°^C) and low salinity (-5 psu).

**FIGURE 6:**
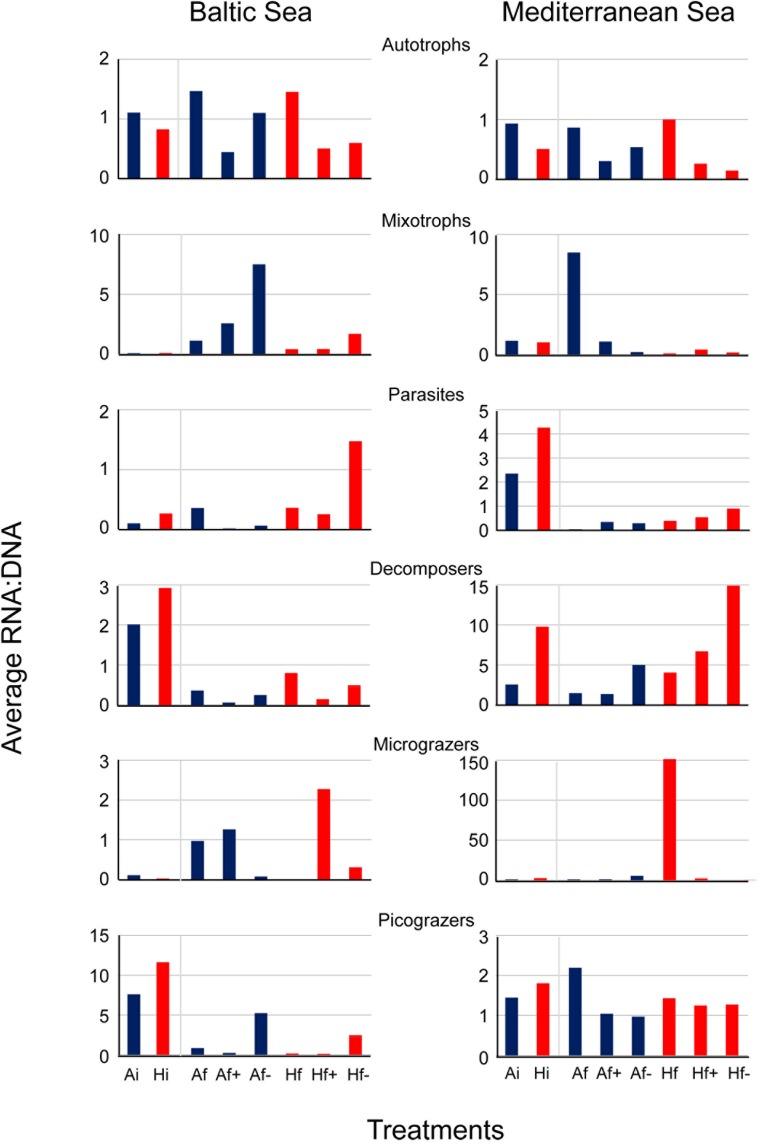
Average RNA:DNA ratios from the 6 trophic groups in the Baltic experiment and in the Mediterranean experiment. A_i_, inoculum and ambient temperature; A_f_, ambient temperature and salinity; A_f+_, ambient temperature and high salinity (+5 psu); A_f-_, ambient temperature and low salinity (-5 psu); H_i_, inoculum and heat shock (+6^°^C); H_f_, heat shock (+6^°^C) and ambient salinity; H_f+_, heat shock (+6^°^C) and high salinity (+5 psu); H_f-_, heat shock (+6^°^C) and low salinity (-5 psu).

In the Baltic experiment, similar rRNA:rDNA ratios were observed for Bacillariophyceae in A_i_ and H_i_ but reduced ratios were observed in A_f+_ and A_f-_ in comparison with A_f_ and H_f+_ and H_f-_ in comparison with H_f_ (**Figure 5**). The ratios for autotrophs were similar to Bacillariophyce including the decrease after salinity changes (**Figure 6**). In the Mediterranean experiment, increased rRNA:rDNA ratio was observed for Dinophyceae in H_i_ community compared to A_i_ community and decreased ratios in A_f+_ and A_f-_ in comparison with A_f_ and H_f+_ and H_f-_ in comparison with H_f_ (**Figure 5**). The highest rRNA:rDNA ratios in the Baltic experiment were observed for Telonemia in A_f+_ and H_f+._ In the Mediterranean experiment, the highest rRNA:rDNA ratio was observed for Fungi in H_f-_ whose rRNA:rDNA ratios increased with salinity changes in both communities (**Figure 5**).

### Resistance

In the Baltic experiment, OTU-richness responded more negatively to salinity increase (A_f+_, H_f+_), while the temperature pre-treatment had little effect on resistance (**Table 2**). In the Mediterranean only A_f-_ showed a clear negative response of OTU-richness to salinity decrease, while all other effects remained small. The negative response of evenness in the Baltic experiment was reversed compared to S, being stronger in A_f-_ and H_f-_. In the Mediterranean experiment, evenness remained relatively resistant only in the case of A_f+_, while all other treatments showed a marked decrease. The two trophic structure indicators (S_auto_/S_het_ and S_auto_/S_grazers+parasites_ ratios) behaved similarly, with a strong increase in the pre-heated H_f+_ and H_f-_ in the Baltic experiment and in A_f+_ and H_f+_ in the Mediterranean experiment.

**Table 2:** Log response ratio (LR) according to OTUs richness, evenness and S_auto_/S_het_ and S_auto_/S_grazers + parasites_ ratios in each experiment.

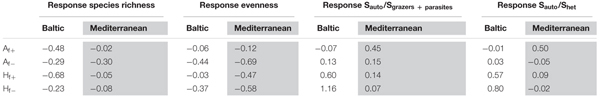

*Values for the Mediterranean experiment are shaded.*

## Discussion

Our experimental results using sequential stressors, heat shock and salinity changes, demonstrate strong effects on OTU richness, community taxonomic and functional composition and rRNA:rDNA ratio of the Baltic and the Mediterranean plankton unicellular eukaryotes. We observed loss of OTUs together with significant compositional changes after the heat shock. At the same time increased rRNA:rDNA ratios for heterotrophic related OTUs were observed. Very few shared OTUs were found across the salinity treatments in each community; in the Baltic experiment reduced OTU richness were observed under high and low salinity while in the Mediterranean experiment under low salinity. We also found high variability on the response of rRNA:rDNA ratios after salinity changes among the taxonomic and trophic groups. The different effect of heating on the resistance to salinity changes between the Baltic and the Mediterranean experiments highlights the importance of the diverse species co-tolerances *sensu* Vinebrooke et al. (2004).

### Temperature and Salinity Changes Impact on Community Composition

Our results show that heat shock lead to significantly different compositional communities which further shifted after salinity changes in both experimental sites supporting the first part of hypothesis 1. Temperature increase is known to influence unicellular planktic communities and food web structure (Moran et al., 2010; Lewandowska and Sommer, 2010; Moustaka-Gouni et al., 2016). In our experiments we found that the H_i_ community in both experiments included more of smaller unicellular primary producers in comparison with the A_i_ community as indicated by increased DNA-based OTUs of nanoplanktic Haptophyceae and picoplanktic Chlorophyceae together with decreased DNA-based OTUs of large diatoms and dinoflagellates. This shift toward smaller phytoplankters due to higher temperatures is seen as a manifestation of Bergmann’s rule and has been found both at the global biogeographic scale and in individual experiments (reviewed by Sommer et al., 2016). While being less diverse in autotrophic OTUs, H_i_ communities were richer in heterotrophic OTUs. Temperature comprises a major environmental factor controlling the metabolic rates of organisms (Brown et al., 2004) and several studies have indicated that heterotrophic processes are highly temperature dependent and are intensified by increased temperature (Lewandowska and Sommer, 2010; López-Urrutia et al., 2006). Indirect beneficial effects of the heat shock might have resulted from cell lysis of sensitive taxa and subsequent DOC-release (Wohlers et al., 2009) and, therefore, stimulated bacterial growth and provided food for grazers of bacteria (Moustaka-Gouni et al., 2016). These could be possible explanations for the compositional shifts toward unicellular heterotrophic flagellates at elevated temperatures. The very few shared DNA-based OTUs across the three salinity treatments were associated mainly with 4 taxonomic groups (Bacillariophyceae, Dinophyceae, Chlorophyceae, Fungi) and specifically the genera *Skeletonema*, *Chaetoceros, Prorocentrum, Gyrodinium*, *Gymnodinium*, *Desmochloris, Kappamyces* and several genera of the class Exobasidiomycetes. These genera are known for their euryhaline distribution *in situ*; several species of *Chaetoceros* have been recorded from estuaries (Terada and Ichimura, 1979) to hypersaline coastal lagoons (Gilabert, 2001). *Skeletonema* grows well between salinities of 10 and 35 psu (Balzano et al., 2011) and *Prorocentrum* between salinities of 20 to 40 psu (Xu et al., 2010). The aforementioned ranges included the salinity range of both experiments. Also, several *Gyrodynium* and *Gymnodinium* species have been characterized as euryhaline and formed red tides under different hydrographic conditions (Zingone and Enevoldsen, 2000; Nagasoe et al., 2006). The rarely studied green alga *Desmochloris* grows well both in freshwater and marine media (Darienko et al., 2009). *Kappamyces* and several genera of the class Exobasidiomycetes were recorded in several marine environments with different salinities across Europe (Richards et al., 2015). However, the high number of specific OTUs recorded in each of the three salinity levels demonstrated the strong salinity effects on biodiversity confirming that salinity can play a crucial role for differential survival of species (Logares et al., 2009). Besides, it is known that species even within the same taxonomic group exhibit different salinity tolerance (Brand, 1984; Park and Simpson, 2010). We have to add one caveat to the comparison with field distributions. Statements about tolerance ranges in our experiments are based on the standing genetic variation within small plankton samples, while tolerance ranges inferred from biogeographic distribution result from the global range of genetic variation of a taxon.

The decrease in OTU richness in H_i_ in comparison with A_i_ is in accordance with the second part of hypothesis 1 and parallels previous findings of negative effects of temperature increase on morpho-species richness in marine environments (Gruner et al., 2017) which has been suggested to result from faster species replacement under warmer conditions (Burgmer and Hillebrand, 2011) combined with the exclusion of invasions by new species in a closed experimental system. The reduced OTU richness after salinity changes in the Baltic experiment and under low salinity in the Mediterranean experiment is in agreement with the second part of hypothesis 1 and can be explained by cell physiology. This richness decrease was not observed under high salinity in the Mediterranean experiment for which we found a higher OTU richness both in A and H communities. Particularly, increased salinities positively affected the MAST and ciliophoran richness. The specific Mediterranean experiment site is influenced by riverine freshwater fluxes leading to a decreasing trend in salinity the last decade (Krestenitis et al., 2012). This recent salinity lowering in a generally high salinity marine environment of the Mediterranean might negatively affect the survival of Mediterranean marine protists while the experimentally increased salinities gave these organisms the opportunity to increase their biodiversity.

### Temperature and Salinity Changes Impact on Community Relative Abundance and Inferred Activity

The high percentages of rare OTUs (>75%) during the experiments match those of the majority of studies on marine protists (Stoeck et al., 2010; Logares et al., 2014). It has been hypothesized that rare marine microbes include ecologically redundant taxa that could increase in abundance following environmental changes and maintain continuous ecosystem functioning (Logares et al., 2015). In the Baltic experiment rare decomposers (*Tolypocladium*, *Cladosporium*, *Thraustochytriaceae*) became abundant after salinity changes and in the Mediterranean experiment picograzers rare at ambient salinity (Centroheliozoa, *Caecitelus, Blepharisma*) became abundant at high and low salinity. Our data showed a positive correlation between rRNA and rDNA relative abundance in both experiments in agreement with previous findings for marine protists (Logares et al., 2014). Contrary to marine bacteria that commonly present disproportionately low activity due to dormancy (Campbell et al., 2011), marine protist activity is usually positively correlated to their abundance (Logares et al., 2014).

The majority of the taxonomic and trophic groups in our experiments had increased or decreased rRNA:rDNA between A_i_ and H_i_ communities and after salinity changes in accordance with hypothesis 2. However, using rRNA to distinguish active and non-active organisms has been criticized for its reliability (Blazewicz et al., 2013). The high variation of the overall range of rRNA:rDNA ratio for Amoebozoa, Apusozoa, Hacrobia and Fungi suggests that the activity of these taxonomic groups was affected more by increased temperature and salinity changes than the activity of Bacillariophyceae, Dinophyceae and MALVs which showed little changes of rRNA:rDNA across the treatments. The increased rRNA:rDNA ratios of Amoebozoa, Apuzosoa and MAST and at the same time of micrograzers and picograzers in H_i_ community in comparison with A_i_ can be attributed to more intensive heterotrophic processes at elevated temperatures. The remarkably increased rRNA:rDNA ratios for Haptophyceae and Telonemia under elevated salinity in the Baltic experiment (21.4 psu) may be coupled with their preference of high salinity Baltic Sea’s microenvironments (from 18 to 22 psu) (Hu Y. et al., 2016) in conjunction with the availability of prey. Higher bacterial abundances and productivity have been also recorded in higher salinity Baltic Sea microenvironments in comparison with fresher microenvironments (Lindh et al., 2015). Finally, the increased rRNA:rDNA ratios in low salinity treatments for Fungi in the Mediterranean experiment (28.8 psu) can be explained by the accumulated dead matter observed under microscopy of samples (data not shown). Fungi have the role of decomposers in marine ecosystems (Richards et al., 2015) and their increased rRNA:rDNA ratios coincided with the reduced total OTU richness.

### Resistance

Neither hypothesis 3 (decreased resistance after the heat-shock) nor its opposite (increased resistance) were unequivocally supported by the calculated response according to the four selected metrics (OTUs richness, evenness, S_auto_/S_het_ and S_auto_/S_grazers + parasites_ ratios), except for the trophic state indicators in the Baltic experiment and to some extent by evenness in the Mediterranean experiment (true for salinity increase, false for salinity decrease). It is a longstanding assumption that biodiversity loss affects ecosystem function and decreases the resistance and the maintenance of ecosystem processes against environmental alterations (Loreau et al., 2001). The coexistence of many species in more biodiverse ecosystems provides a greater guarantee that some will back up when others fail after environmental disturbances (Wittebolle et al., 2009). In the Mediterranean experiment, we noticed a support of the above assumptions since the increased temperature-induced biodiversity loss in the H_i_ community lead to lower resistance against salinity changes. According to the model of Vinebrooke et al. (2004) this would result from a negative correlation between heat and salinity tolerances of the OTUs. The genetically less biodiverse H_i_ community lead salinity treatments to display greater change from initial community via greater species loss and consequently had lower resistance against increased/decreased salinity. Besides, it has been found that genetically less diverse communities have lower chances to contain taxa with complementary response traits and the ability for rapid compensatory growth after a disturbance which may decrease resistance (Shade et al., 2012). In our experiment, the nature of change (salinity increase vs. decrease), the choice of the response parameter and the geographic origin of the experimental community were at least as influential on resistance as diversity loss in response to the temperature pre-treatment. Both plankton communities originate from coastal ecosystems, where salinity changes are frequent. Thus, experimental communities might contain residuals of species adapted to higher or lower salinities than *in situ* ones, depending on the history of the local community. In the case of the heterotrophic OTUs, the autecological response to salinity change may be superseded by creation of additional niches by the death of non-resistant OTUs with subsequent detritus and DOC production, which fuels bacterial growth and thereby improves the feeding conditions for bacterivores.

## Conclusion

In this study we examined the influence of biodiversity changes caused by an intermittent heat-shock on the sensitivity of plankton community to subsequent salinity changes. Our experiment provided evidence that the effect of the initial environmental stressor (heat wave) on plankton community resistance to the subsequent stressor (salinity changes) is contingent on the local species pool and the resultant patterns of species co-tolerance (*sensu* Vinebrooke et al., 2004). Identically designed mesocosm experiments were performed in the Baltic Sea where diatoms dominated in plankton DNA and RNA dataset and in the Mediterranean Sea where dinoflagellates were the dominant plankton OTUs. In the Baltic experiment, the less diverse heat shocked plankton communities showed a higher resistance to salinity changes in apparent contradiction to the assumption, that biodiversity should provide an insurance of ecosystem functions against environmental disturbances (Loreau et al., 2001). On the contrary, in the Mediterranean experiment the assumption that biodiversity acts as a buffer against environmental fluctuations was supported and the induced less biodiverse heated shocked community had lower resistance to salinity changes. The frequent strong environmental fluctuations (including salinity changes) in the Baltic Sea region most probably played a key role on the relatively increased resistance against the two stressors (heat-shock and salinity changes). On the other hand, the less exposed Mediterranean plankton community to environmental fluctuations had as a consequence the increased sensitivity, mainly in the heated community, against salinity alterations.

## Author Contributions

NS, US, and MM-G designed the experiment. NS and SG carried out the molecular, bioinformatics, and statistical analysis. NS carried out the experiment prepared the manuscript. NS, JL-B, US, and MM-G designed the research. NS, SG, JL-B, US, and MM-G revised the manuscript.

## Conflict of Interest Statement

The authors declare that the research was conducted in the absence of any commercial or financial relationships that could be construed as a potential conflict of interest.

## Footnotes

1 http://mrdnalab.com/

2 www.mrdnalab.com
