# Supplementary material for: Unicellular Eukaryotic Community Response to Temperature and Salinity Variation in Mesocosm Experiments
Source: Front Microbiol. 2018 Oct 9;9:2444. doi: 10.3389/fmicb.2018.02444 (PMC6189394; doi:10.3389/fmicb.2018.02444)
Supplement: Supplementary file 1 [file Data_Sheet_1.PDF]

Unicellular eukaryotic community response to temperature and salinity variation in mesocosm experiments

Natassa Stefanidou[1], Savvas Genitsaris[1,2], Juan Lopez-Bautista[3], Ulrich Sommer[4*] and Maria Moustaka-Gouni[1*]

[1]Department of Botany, School of Biology, Aristotle University of Thessaloniki, 54124 Thessaloniki, Greece

[2]School of Economics, Business Administration & Legal Studies, International Hellenic University, 57001 Thermi, Greece

[3]Department of Biological Sciences, The University of Alabama, P.O. Box. 870345, Tuscaloosa, AL 35484-0345, USA

[4]Geomar Helmholtz Centre for Ocean Research Kiel, 24105, Kiel, Germany

*Corresponding Authors, Email addresses: usommer@geomar.de, mmustaka@bio.auth.gr

Keywords: coastal marine plankton, climate change, Illumina sequencing, 18S rRNA gene, cDNA, RNA/DNA ratio

Supplementary Tables

Supplementary Table 1: Annotated major trophic role and DNA-based and RNA-based OTUs number. Values for the Mediterranean experiment are shaded.

Super groups	Groups	Class	Number of OTUs				Trophic role	References
			DNA	RNA	DNA	RNA		
Alveolates	Apicomplexa		2	2	3	3	Parasites	Skovgaard, 2014
	Ciliophora	Colpodea	0	1	0	1	Nano-grazers	
		Heterotrichea	1	1	5	1	Nano-grazers	
		Litostomatea	2	8	3	5	Parasites	
		Oligohymenophorea	1	7	12	4	Nano-grazers/ Parasites	Lynn, 2008
		Phyllopharyngea	8	23	4	5	Nano-grazers/ Parasites	
		Prostomatea	0	5	1	2	Nano-grazers	
		Spirotrichea	20	70	99	53	Nano-grazers	
	Dinophyceae		125	50	280	103	Micro-grazers/ Mixotrophs	Sherr and Sherr, 2007; Hansen 2011
	MALVs		13	9	38	21	Parasites	Guillou et al., 2008
Amoebozoa	Lobosa		3	7	4	18	Micro-grazers	Cavallier- Smith et al., 2006
Apusozoa	Hilomonadea		1	6	2	3	Pico-grazers	Scheckenbach et al., 2006
Archaeplastida	Chlorophyta		81	46	59	67	Autotrophs	Not et al., 2012
	Rhodophyta		1	1	2	3	Autotrophs	Yokoyama et al., 2009
Excavata	Discoba		1	1	1	1	Pico-grazers	Cavallier- Smith et al., 2006
Harcobia	Centroheliozoa		4	4	10	31	Pico-grazers	Burki et al., 2009
	Cryptophyta		0	1	3	1	Autotrophs/Mixotrophs	Not et al., 2012
	Haptophyta		38	92	31	33	Autotrophs/Mixotrophs	Not et al., 2012
	Katablepharidophyta		2	4	1	0	Pico-grazers	Okamoto et al., 2005
	Picobiliphyta		17	4	8	6	Pico-grazers	Moreira et al., 2014
	Telonemia		7	53	6	7	Pico-grazers	Boenigk et al., 2002
Opisthokonta	Choanoflagellida			25	39	42	Pico-grazers	
	Fungi	Ascomycota	39	55	42	130	Decomposers	
		Basidiomycota	36	58	48	90	Decomposers	
		Blastocladiomycota	0	1	1	0	Parasites	Richards et al., 2015
		Chytridiomycota	14	16	16	15	Parasites	
		Cryptomycota	5	2	4	1	Decomposers	
		Entomophthoromycota	1	4	1	4	Decomposers	
		Mucoromycota	2	4	2	6	Decomposers	
	Mesomycetozoa		3	9	11	22	Parasites	Marshall et al., 2008
Rhizaria	Cercozoa		71	56	40	41	Parasites	Chantangsi et al., 2010
Stramenopiles	Bacillariophyta		201	230	94	45	Autotrophs	Not et al., 2012
	Bicoecea		30	21	49	46	Pico-grazers	Boenigk et al., 2002
	Bolidophyceae and relatives		10	12	3	3	Autotrophs	Brown and Sorhannus, 2010
	Chrysophyceae-Synurophyceae		6	29	4	8	Autotrophs/Mixotrophs	Brown and Sorhannus, 2010
	Dictyochophyceae		15	12	10	6	Autotrophs	Brown and

							Sorhannus, 2010
	Eustigmatophyceae	2	1	0	4	Autotrophs	Brown and Sorhannus, 2010
	Hyphochytriomyceta	0	0	3	1	Parasites	Kramarsky-Winter et al., 2006
	Labyrinthulea	22	20	108	38	Parasites	Raghukumar, 2002
	MAST	60	29	27	15	Pico-grazers	Massana et al., 2004
	Oomyceta	11	10	11	8	Parasites	Park et al., 2004
	Pelagophyceae	1	2	2	3	Autotrophs	Brown and Sorhannus, 2010
	Phaeophyceae	3	0	0	1	Autotrophs	Brown and Sorhannus, 2010
	Picophagea	1	2	1	1	Mixotrophs	Cavallier- Smith et al., 2006
	Pirsonia	3	4	4	14	Parasites	Skovgaard, 2014
	Raphidophyceae	2	1	1	0	Autotrophs	Brown and Sorhannus, 2010

Supplementary Table 2A: Total number of DNA-based OTUs and RNA-based reads of major taxonomic groups for each experiment. Values for the Mediterranean experiment are shaded.

		Baltic		Mediterranean	
		**DNA OTUs**	**RNA Reads**	**DNA OTUs**	**RNA Reads**
Alveolates	Dinophyceae	248	3824	403	48784
	MALVs	31	123	60	1565
	Other Alveolates	90	64491	207	76590
Archeoplastida	Chlorophytes	139	2268	123	10039
Harcobia	Haptophytes	53	74696	64	21723
	Other Harcobia	82	16445	75	16593
Opisthoconta	Fungi	275	25128	239	182643
	Other Opisthoc.	49	2732	109	16073
Rhizaria	Cercozoa	117	6774	81	3473
Stramenopiles	Bacillariophyceae	382	289044	178	16905
	Other Stramen.	360	24775	426	83368
Amoebozoa	Lobosa	3	1160	5	1416
Apusozoa	Hilomonadea	5	1127	4	189
Excavata	Discoba	4	10	1	2

Supplementary Table 2B: Total number of DNA-based OTUs and RNA-based reads of trophic groups for each experiment. Values for the Mediterranean experiment are shaded.

	Baltic		Mediterranean	
	**DNA**	**RNA**	**DNA**	**RNA**
Autotrophs	374	304766	129	76153
Mixotrophs	40	74443	298	21268
Decomposers	97	25128	114	180643
Parasites	124	9077	212	43156
Micrograzers	67	3486	70	7413
Picograzers	166	95264	270	149458

Supplementary Table 3: Ratios of auto- and mixo- trophic OTUs to heterotrophic OTUs (S_{auto}/S_{het} ratio) and auto- and mixo- trophic OTUs to consumers ($S_{auto}/S_{grazers+parasites}$ ratio) for each experiment. Values for the Mediterranean experiment are shaded.

Baltic	Mediterranean

	$S_{auto}/S_{grazers+parasites}$	S_{auto}/S_{het}	$S_{auto}/S_{grazers+parasites}$	S_{auto}/S_{het}
A_i	1.48	1.13	1.43	1.10
A_f	1.23	0.92	0.97	0.74
A_{f+}	1.17	0.91	1.49	1.21
A_{f-}	1.41	0.95	1.13	0.70
H_i	1.34	0.89	0.52	0.41
H_f	0.62	0.46	0.58	0.50
H_{f+}	1.13	0.81	0.67	0.55
H_{f-}	1.99	1.01	0.62	0.49

Supplementary Table 4: Percentages of DNA-based and RNA-based abundant and rare OTUs for each experiment. Values for the Mediterranean experiment are shaded.

	**DNA**				**RNA**			
	**Abundant OTUs %**		**Rare OTUs %**		**Abundant OTUs %**		**Rare OTUs %**	
	**Baltic**	**Mediterranean**	**Baltic**	**Mediterranean**	**Baltic**	**Mediterranean**	**Baltic**	**Mediterranean**
A_i	3.7	4.6	87.6	82.7	3.3	4.9	92.0	82.3
A_f	5.3	10.4	88.4	85.4	3.2	6.5	93.0	82.1
A_{f+}	10.2	10.9	84.2	77.4	2.3	5.3	94.5	82.6
A_{f-}	8.0	8.9	84.5	81.7	2.8	6.3	91.5	86.2
H_i	7.6	3.3	83.9	91.7	3.3	3.5	88.0	88.8
H_f	4.1	5.2	86.8	87.4	1.5	6.5	88.7	81.3
H_{f+}	5.9	6.9	85.2	85.6	4.6	7.7	90.8	79.5
H_{f-}	3.8	5.9	86.7	87.5	1.9	8.3	90.3	79.4

References

Boenigk, J., Arndt, H. (2002). Bacterivory by heterotrophic flagellates: community structure and feeding strategies. Anton van Lee 81, 465-480.

Brown, J.W., Sorhannus, U. (2010). A molecular genetic timescale for the diversification of autotrophic Stramenopiles (Ochrophyta): substantive underestimation of putative fossil ages. PLoS ONE 5 (9), e12759.

Burki, F., Inagaki, Y., Bråte, J., Archibald, J.M., Keeling, P. J., Cavalier-Smith, T. et al. (2009). Large-scale phylogenetic analyses reveal that two enigmatic protist lineages, Telonemia and Centroheliozoa, are related to photosynthetic Chromalveolates. Genome Biol Evol 1, 231-238.

Cavallier- Smith, T., Chao, E.Y.E. (2006). Phylogeny and Megasystematics of Phagotrophic Heterokonts (Kingdom Chromista). J Mol Evol 62, 388-420.

Chantangsi, C., Leander, B.S. (2010). An SSU rDNA barcoding approach to the diversity of marine interstitial cercozoans, including descriptions of four novel genera and nine novel species. Int J Syst Evol Microbiol 60, 1962-1977.

Guillou, L., Viprey, M., Chambouvet, A., Welsh, R.M., Kirkham, A.R., Massana, R., et al. (2008). Widespread occurrence and genetic diversity of marine parasitoids belonging to Syndiniales (Alveolata). Environ Microbiol 10, 3349-3365.

Hansen, P.J. (2011). The Role of Photosynthesis and Food Uptake for the Growth of Marine Mixotrophic Dinoflagellates. Eukaryot Microbiol 58, 203-214.

Kramarsky-Winter, E., Harel, M., Siboni, N., Ben Dov, E. Brickner, I., Loya, Y. et al. (2006). Identification of a protist-coral association and its possible ecological role. MEPS 317: 67-73.

Lynn, D. (2008). The ciliated protozoa: characterization, classification, and guide to the literature. New York: Springer.

Marshall, W.L., Celio, G., McLaughlin, D.J., Berbee, M.L. (2008). Multiple isolations of a culturable, motile Ichthyosporean (Mesomycetozoa, Opisthokonta), Creolimax fragrantissima n. gen., n. sp., from marine invertabrate digestive tracts. Protist 159, 415-433.

Massana, R., Castresana, J., Balagué, V., Guillou, L., Romari, K., Groisillier, A. et al. (2004). Phylogenetic and Ecological Analysis of Novel Marine Stramenopiles. Appl Environ Microbiol 70 (6), 3528-3534.

Moreira, D. and López-García, P. (2014). The rise and fall of Picobiliphytes: How assumed autotrophs turned out to be heterotrophs. BioEssays 36, 468–474.

Not, F., Siano, R., Kooistra, W.H.C.F., Simon, N., Vaulot, D., Probert, I. (2012). Diversity and ecology of eukaryotic marine phytoplankton. In: Genomic insights into the biology of algae. Piganeau, ed. USA: Elsevier Chapter One, pp. 1-54.

Okamoto, N., Inouyen, I. (2005). The Katablepharids are a Distant Sister Group of the Cryptophyta: A Proposal for Katablepharidophyta Divisio Nova/Kathablepharida Phylum Novum Based on SSU rDNA and Beta-Tubulin Phylogeny. Protist 156, 163-179.

Park, M.G., Yih, W., Coats, D.W. (2004). Parasites and phytoplankton, with special emphasis on Dinoflagellate infections. *J Eukaryot Microbiol* 51, 45-155.

Raghukumar, S. (2002). Ecology of the marine protists, the Labyrinthulomycetes (Thraustochytrids and Labyrinthulids*)*. Eur J Protistol 38, 127-145.

Richards, T.A., Leonard, G., Mahé, F., del Campo, J., Romac, S., Jones, M.D.M. et al. (2015). Molecular diversity and distribution of marine fungi across 130 European environmental samples. Proc R Soc B 282, 2015-2243.

Scheckenbach, F., Wylezich, C., Mylnikov, P.A., Weitere, M., Arndt, H. (2006). Molecular Comparisons of Freshwater and Marine Isolates of the Same Morphospecies of Heterotrophic Flagellates. Appl Environ Microbiol 72 (10), 6638-6643.

Sherr, B.F., Sherr, E.B., Caron, D.A., Vaulot, D., Worden, A.Z. (2007). Oceanic protists. Oceanography 20, 130-134.

Skovgaard, A. (2014). Dirty tricks in the plankton: diversity and role of marine parasitic protists. Acta Protozool 53, 51-62.

Yokoyama, A., Scott, J.L., Zuccarello, G.C., Kajikawa, M., Hara, Y., West, J.A. (2009). Corynoplastis japonica gen. et sp. nov. and Dixoniellales ord. nov. (Rhodellophyceae, Rhodophyta) based on morphological and molecular evidence. Phycol Res 57, 278-289.

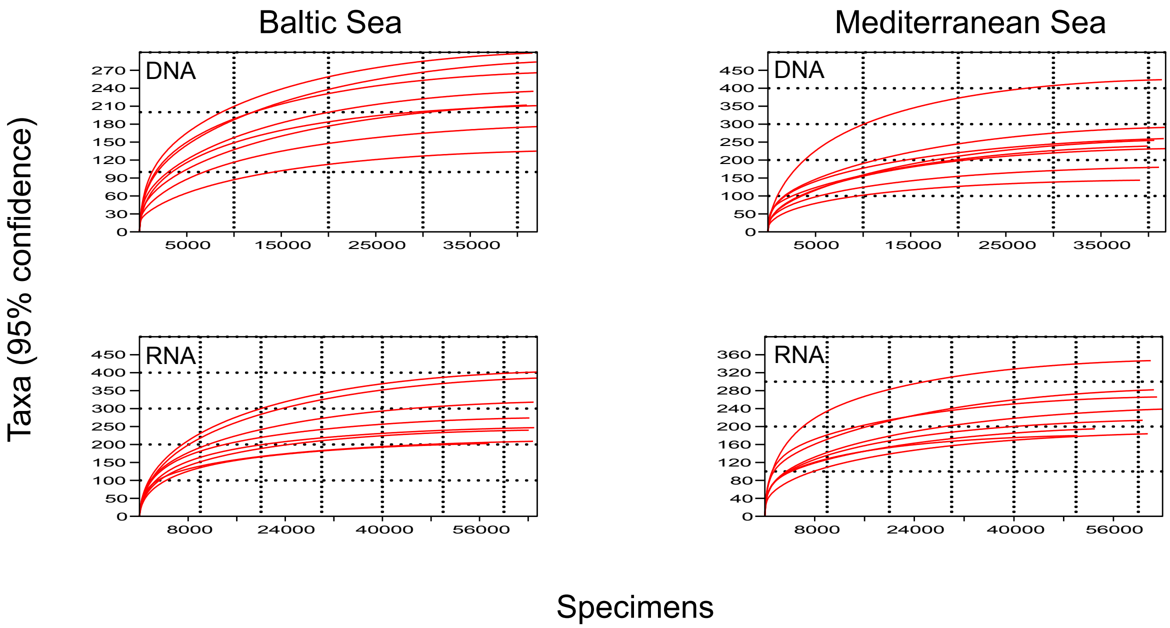

Supplementary Figure 1. Rarefaction curves of DNA and RNA samples in the Baltic experiment and in the Mediterranean experiment.

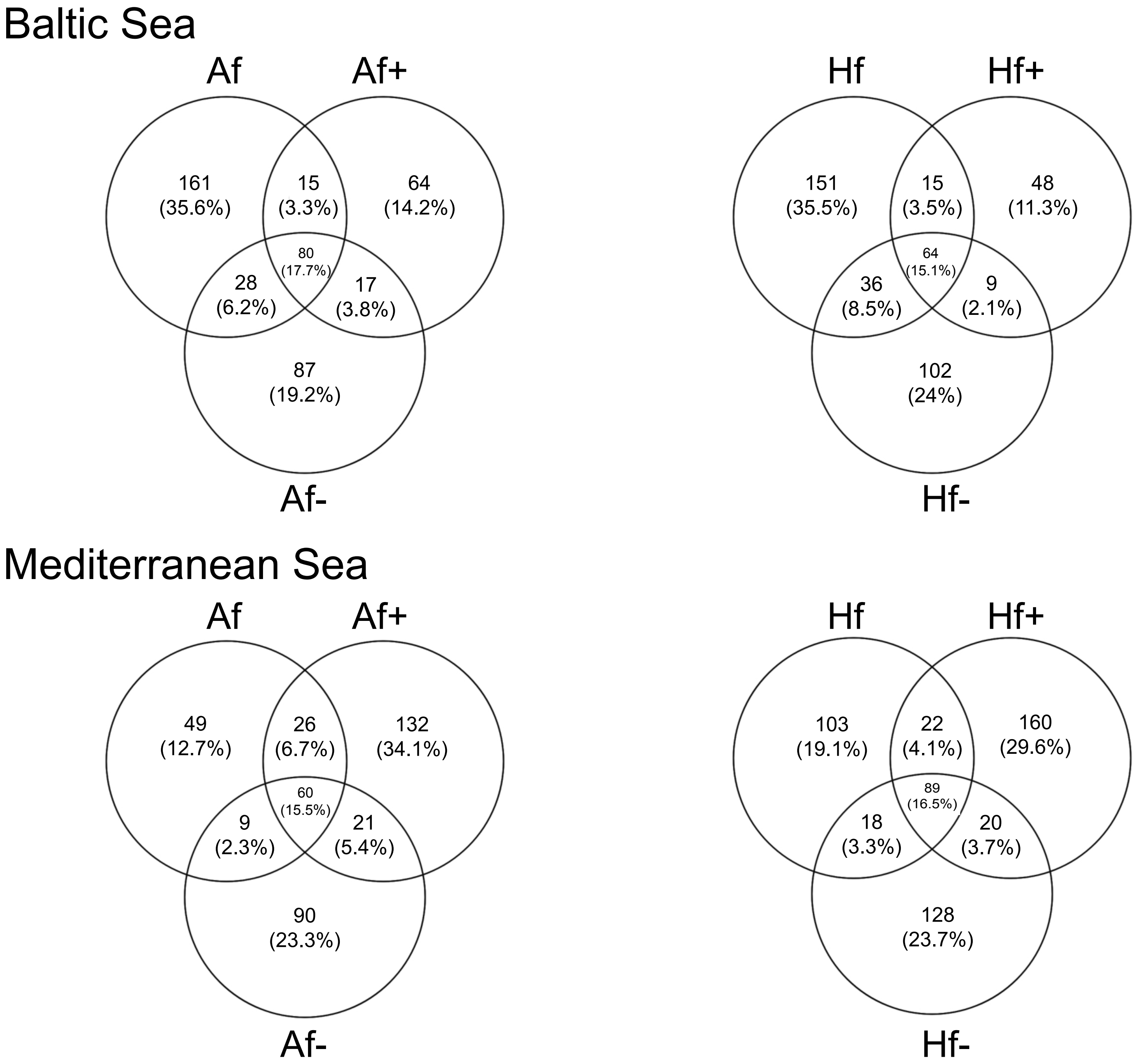

Supplementary Figure 2. Venn diagram showing the number of unique and shared DNA OTUs across the different salinities of A and H communities in the Baltic experiment and in the Mediterranean experiment. Af: ambient temperature and salinity, Af+: ambient temperature and high salinity (+5psu), Af-: ambient temperature and low salinity (-5psu), Hf: heat shock (+6oC) and ambient salinity Hf+: heat shock (+6oC) and high salinity (+5psu), Hf-: heat shock (+6oC) and low salinity (-5psu).

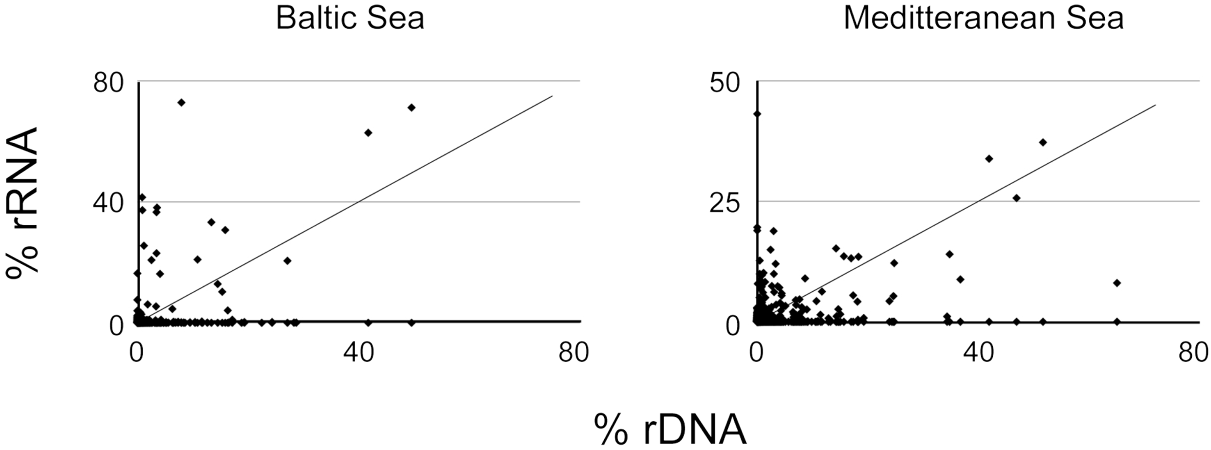

Supplementary Figure 3. Relative read abundance of RNA (y axis) and DNA (x axis) reads in the Baltic experiment and in the Mediterranean experiment. Data points represent the common OTUs in RNA and DNA datasets.

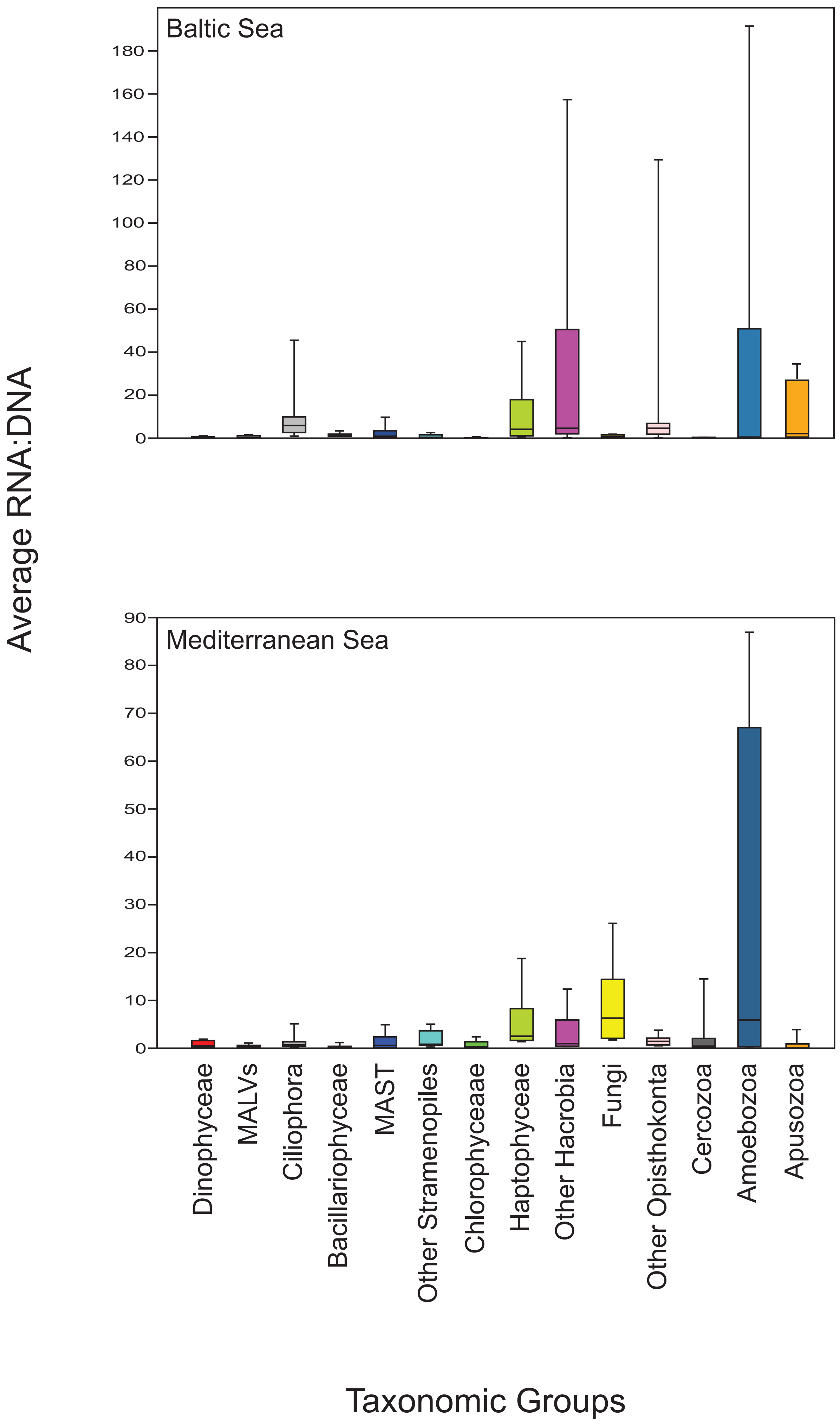

Supplementary Figure 4. Range of average RNA:DNA ratios for each taxonomic group in the Baltic experiment and in the Mediterranean experiment. Boxplots represent variation among the average RNA:DNA ratios in each treatment and whiskers denote minimum and maximum values.
